# Identification of the most suitable reference gene for gene expression studies with development and abiotic stress response in *Bromus sterilis*

**DOI:** 10.1038/s41598-021-92780-1

**Published:** 2021-06-28

**Authors:** Madhab Kumar Sen, Kateřina Hamouzová, Pavlina Košnarová, Amit Roy, Josef Soukup

**Affiliations:** 1 Department of Agroecology and Crop Production, Faculty of Agrobiology, Food and Natural Resources, Czech University of Life Sciences Prague, Kamýcká 1176, 165 00 Prague 6, Suchdol, Czech Republic; 2 Faculty of Forestry and Wood Sciences, EXTEMIT-K and EVA.4.0 Unit, Czech University of Life Sciences, Kamýcká 1176, 165 00 Prague 6, Suchdol, Czech Republic

**Keywords:** Plant genetics, Plant molecular biology, Plant stress responses

## Abstract

*Bromus sterilis* is an annual weedy grass, causing high yield losses in winter cereals. Frequent use of herbicides had led to the evolution of herbicide resistance in this species. Mechanisms underlying herbicide resistance in *B. sterilis* must be uncovered because this problem is becoming a global threat. qRT-PCR and the next-generation sequencing technologies can elucidate the resistance mechanisms. Although qRT-PCR can calculate precise fold changes, its preciseness depends on the expression of reference genes. Regardless of stable expression in any given condition, no gene can act as a universal reference gene. Hence, it is necessary to identify the suitable reference gene for each species. To our knowledge, there are no reports on the suitable reference gene in any brome species so far. Thus, in this paper, the stability of eight genes was evaluated using qRT-PCR experiments followed by expression stability ranking via five most commonly used software for reference gene selection. Our findings suggest using a combination of *18S rRNA* and *ACCase* to normalise the qRT-PCR data in *B. sterilis*. Besides, reference genes are also recommended for different experimental conditions. The present study outcomes will facilitate future molecular work in *B. sterilis* and other related grass species.

## Introduction

One of the major plant protection problems encountered by farmers across the globe is regarding weeds. Herbicides have been widely used to manage weeds and magnify the main crop's yield quality and quantity. Despite their success in managing weeds, constant use of similar herbicides has evolved resistance in many weedy species. Owing to its rapid population dynamics and lack of efficient herbicides, barren brome (*Bromus sterilis *L.) has grown into a troublesome weed in winter cereals in many south and north American countries, middle and west Europe^1–3^. Besides the most frequent acetolactate synthase (ALS) and acetyl-CoA carboxylase (ACCase) resistance in Europe (http://www.weedscience.org/Home.aspx), United Kingdom also reported *B. sterilis* resistance against glyphosate in 2019^4^. These results indicate the prerequisite for monitoring more barren brome populations. Gene expression studies have contributed immensely in elucidating the target gene amplification and expression and the over-expression of detoxifying enzyme genes related to herbicide resistance and other abiotic stresses^5,6^. Moreover, with the development of next-generation sequencing technologies, there is a need to validate the expression of a greater number of genes involved in abiotic stresses^7,8^. qRT-PCR is widely used for such comparative gene expression studies. However, the reliability of the qRT-PCR depends on the selection of a stable reference gene.

Compared to the traditional polymerase chain reaction (PCR), quantitative real-time polymerase chain reaction (qRT-PCR) has many advantages like high specificity, rapidity and sensitivity, making it an essential part of comparative expression studies^9,10^. Previously, the relative quantification of gene expression was done either by Northern blot or by reverse transcription-polymerase chain reaction (RT-PCR). The most important limitation of these methods is their inability to detect extremely low expression, resulting in replacing the pre-existing methods with microarrays and qRT-PCR^9,11^. Even though these modern techniques are highly sensitive and can calculate precise fold changes, their preciseness is highly dependent on the expression of a reference gene. Ideally, a reference gene refers to constitutive genes required to maintain the basic cellular functions of an organism. These genes are known to have stable gene expression in all cells under both normal and pathophysiological conditions^12–14^. However, the steps of qRT-PCR are reclined to variations; therefore, to overcome these variations, target gene transcription levels must be normalised to reference genes transcription levels. Any error in selecting a suitable reference gene may lead to misleading results. Hence, selecting a reliable reference gene is necessary for molecular biology-oriented studies^9,14–17^. The most commonly used references genes for normalisation of plant gene expression studies are *ubiquitin* (*UBQ*)*, β-tubulin* (*β-TUB*)*, ribosomal RNA genes* (*18S rRNA* and *25S rRNA*)*, glyceraldehyde-3-phosphate dehydrogenase* (*GAPDH*)*, eukaryotic elongation factor* (*eEF*)*, eukaryotic translation initiation factor 1 (eIF1), actin* (*ACT*)*, acetyl-CoA carboxylase* (*ACCase*) etc^9,18^. Although these genes are known to have a stable expression in any given condition, several studies documented variability in their expression level between species of plants or different stress conditions or developmental stages^19–21^. As no gene can act as a universal reference, it is necessary to systematically select and identify the suitable reference gene for each species^22,23^.

There are no reports of a suitable reference gene in *B. sterilis* or any other brome species. Our study aims to identify a suitable reference gene for gene expression studies in *B. sterilis* (or barren brome). Increasing the number of treatments might lead to more variations in results, which decreases the chance of identifying a suitable reference gene^9^. In this study, we had selected eight common candidate reference genes (*UBQ*, *ACT*, *GAPDH*, *18S rRNA*, *25S rRNA*, *ACCase*, *β-TUB* and *eEF*) identified in *B. sterilis* and evaluated the stability of their gene expression in three developmental stages (two-leaves, three-leaves and four-leaves), two different plant organs (shoots and leaves) and one abiotic stress (drought stress). Among the various severe issues with detrimental effects, climate change has remained a top priority. Global warming has resulted in an increase of air temperature and evapotranspiration, leading to agricultural droughts, affecting both crops and weeds.

Low soil moisture increase the competition for water and nutrients between weeds and the crop, thus making weed management complicated. Some (usually C4) weed species gain profit from this situation. Uptake and translocation of herbicides within the plant is reduced, thereby affecting the efficacy of the applied herbicides. Hence, interest for studies under drought is recently rising^24^, which might require expression studies with several genes of interest. Therefore, drought stress has been included in the present study, and our recommended reference genes will be helpful in future drought-related studies.

The most suitable candidate was selected based on the ranking provided by different widely used statistical software for reference gene analysis (comparative ΔCt, BestKeeper, NormFinder, geNorm and RefFinder). Additionally, the most suitable reference gene was used to validate a herbicide-stress experiment. Thus, our study provides a basis for identifying the suitable reference gene for future gene expression studies in *B. sterilis* and will aid in impending studies on the molecular basis underlying the herbicide resistance in barren brome.

## Results

### Primer efficiency and candidate genes expression

**Figure 1:**
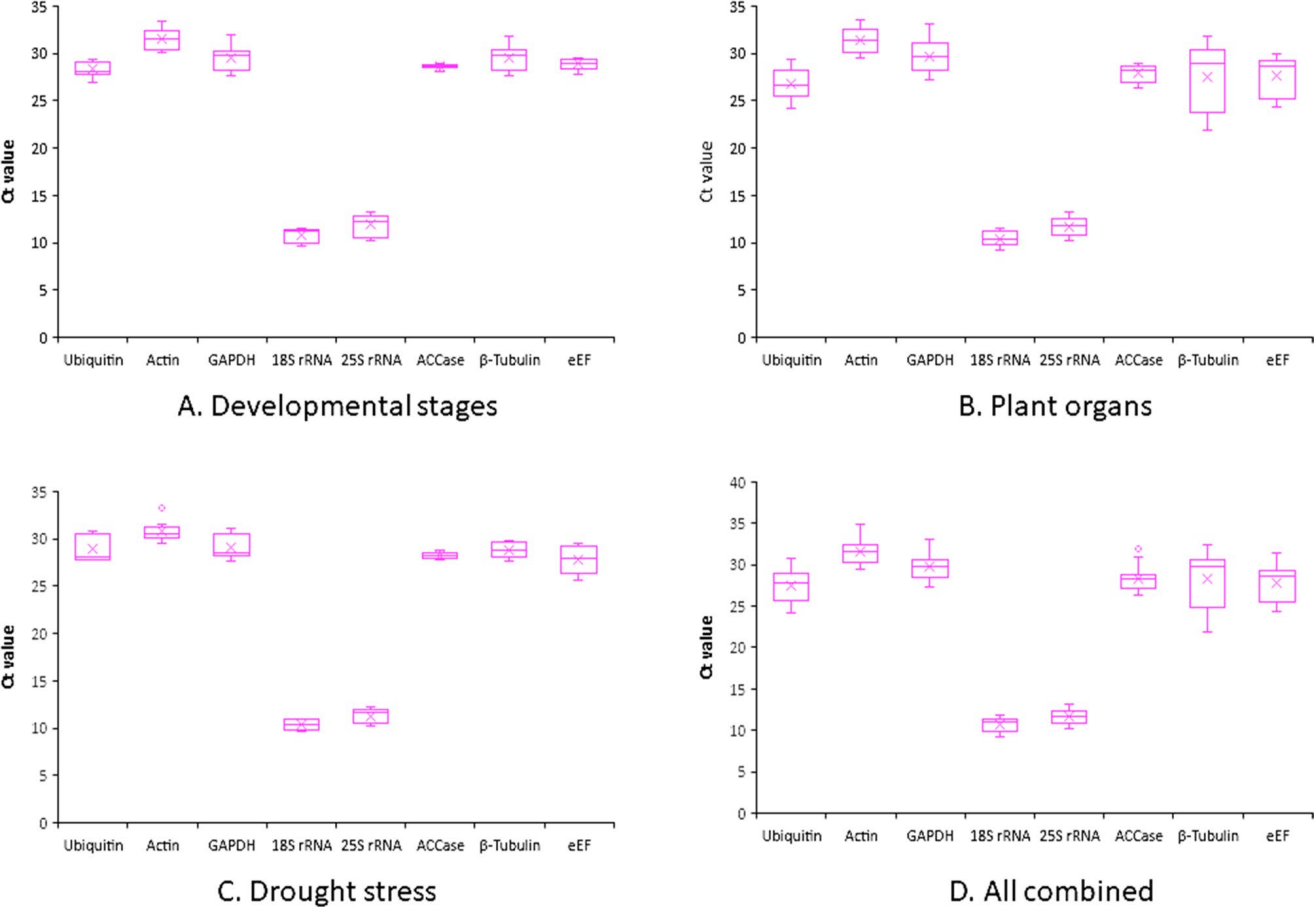
Expression levels of the eight candidate genes. Ct values obtained from three developmental stages (2nd, 3rd and 4th leaves), two different plant organs (shoots and leaves) and abiotic stress (drought stress) were compared and plotted.

1.2% agarose gel electrophoresis was used to check the integrity of the RNA. In addition, the quantity and quality of RNA were evaluated by a nanodrop spectrophotometer (Thermo Scientific™, US). The A260/A280 values ranged from 1.90 to 2.05. These samples were further used to synthesise cDNA, which was used for the qRT-PCR experiments. In all the qRT-PCR amplification, a single peak was obtained (supplementary Fig. 1). The selected primers for this study showed a single band in the 1.5% agarose gel (supplementary Fig. 2) and had efficiency values ranged between 92.32 and 106.79%, which falls under the acceptable range. The correlation coefficient values ranged from 0.980 to 0.999 (Table 1). The expression profile of the 8 candidate genes under different experimental conditions is shown in the Fig. 1. *18s rRNA* showed the lowest cycle threshold value (Ct), indicating high expression of the gene, whereas *ACT* showed the highest Ct value indicating low expression.

**Table 1:** Primer information of the eight candidate reference genes.

Gene	Sequence	Annealing temperature (°C)	Amplicon length (bp)	Primer efficiency (%)	R^2^ value
*Ubiquitin*_forward primer	GCACAAGCACAAGAAGGTGA	60	120	99.46	0.997
*Ubiquitin*_reverse primer	AGTGGTTTGCCATGAAGGTC				
*Actin*_forward primer	ATGCGATTCTTCGTTTGGAC		172	102.34	0.980
*Actin*_reverse primer	GATGTCTCCAGCTCCTGCTC				
*GAPDH*_forward primer	AGCGACATCAAGCTCAAGAA	58	241	92.44	0.994
*GAPDH*_reverse primer	CGTTGACACCAACCACAAAC				
*18S rRNA*_forward primer	AAACGGCTACCACATCCAAG		154	92.42	0.999
*18S rRNA*_reverse primer	CCTCCAATGGATCCTCGTTA				
*25S rRNA*_forward primer	CCCAGTGCTCTGAATGTCAA		211	92.32	0.999
*25S rRNA*_reverse primer	GTCTTCTTTCCCCGCTGATT				
*ACCase*_forward primer	GCTGCTATTGCCAGTGCTTA	57	171	95.77	0.989
*ACCase*_reverse primer	AAGCTTGTTCAGGGCAGAAA				
*β-Tubulin*_forward primer	AGTACCGTGCCCTCACAGTC		150	106.79	0.996
*β-Tubulin*_reverse primer	TCTGCTCGTCAACCTCCTTT				
*eukaryotic elongation factor*_forward primer	CCTGCACTGTCATTGATGCT		185	94.51	0.988
*eukaryotic elongation factor*_reverse primer	CTGCCTGACACCAAGAGTGA

### Gene expression stability analysis

#### Developmental stages-related experiments

**Figure 2:**
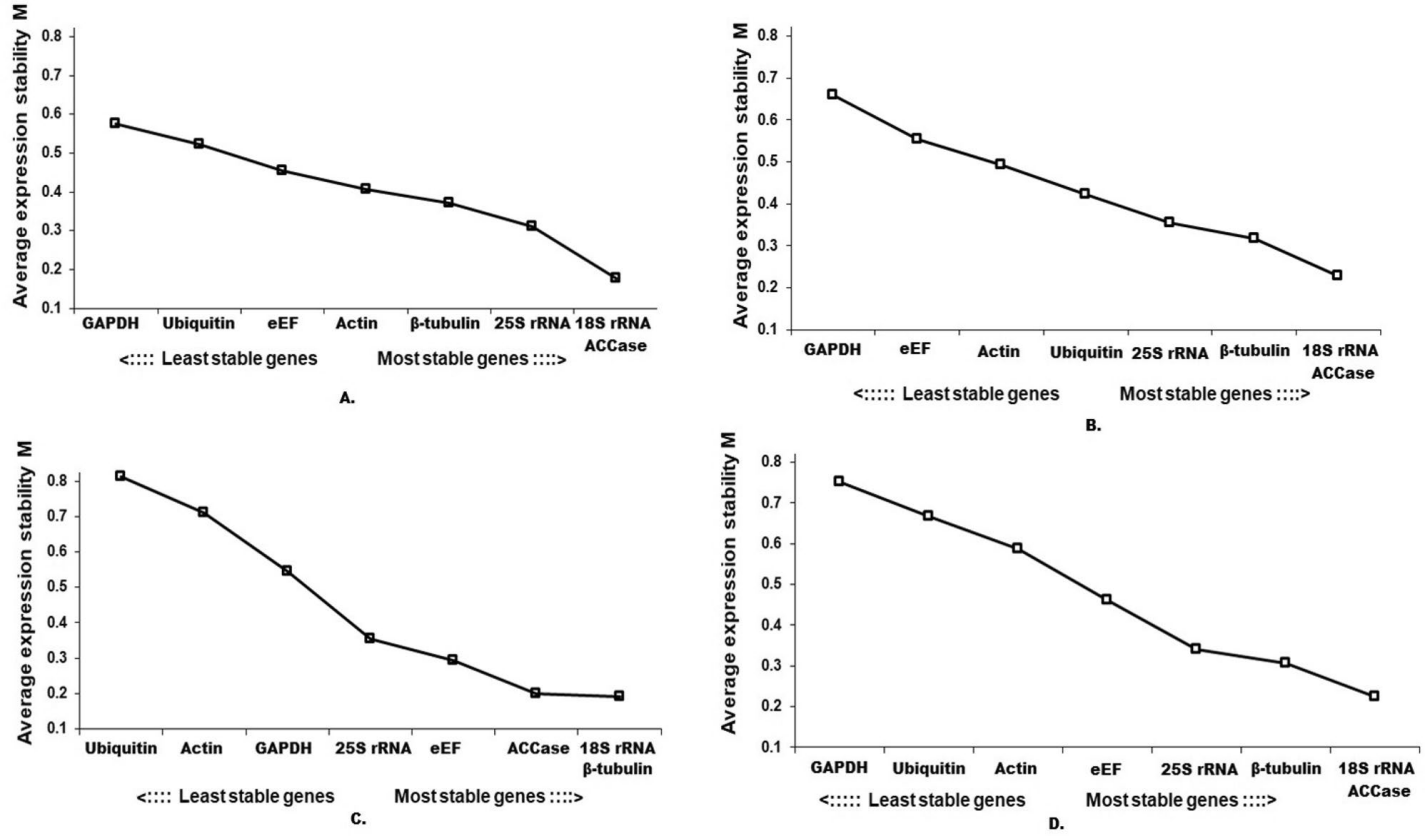
geNorm ranking of the candidate genes under different tested conditions. (**A**) Developmental stages, (**B**) plant organs, (**C**) Drought stress, and (**D**) All combined.

*18S rRNA* was identified as the stable reference gene by comparative ΔCt and RefFinder. BestKeeper software identified *ACCase* as the most stable reference gene (Table 2). NormFinder analysis revealed *18S rRNA* and *eEF* as the most stable genes, whereas geNorm analysis ranked *18S rRNA* and *ACCase* as the best reference gene for developmental stages-related experiments in *B. sterilis* (Table 2, Fig. 2). Except, comparative ΔCt, all the used software identified *GAPDH* as the least stable gene. According to the comparative ΔCt analysis, *eEF* is the least stable gene.

**Table 2:** Expression stability of candidate genes analysed by ΔCt, BestKeeper, NormFinder and RefFinder.

Rank	Comparative ΔCt		BestKeeper		NormFinder		RefFinder	
	Genes	Average of SD	Genes	Std dev [+/− CP]	Gene name	Stability value	Genes	Geomean of ranking values
**Life stages (two-leaves stage, three-leaves stage and four-leaves stage)**								
1	*18S rRNA*	0.7	*ACCase*	0.19	*18S rRNA*	0.132	*18S rRNA*	1.73
2	*Actin*	0.79	*eEF*	0.47	*ACCase*	0.134	*25S rRNA*	2.71
3	*25S rRNA*	0.8	*18S rRNA*	0.65	*Actin*	0.154	*Actin*	2.99
4	*β-tubulin*	0.81	*Ubiquitin*	0.65	*eEF*	0.166	*β-tubulin*	3.25
5	*ACCase*	0.89	*Actin*	0.86	*β-tubulin*	0.215	*ACCase*	3.64
6	*GAPDH*	0.94	*25S rRNA*	0.88	*25S rRNA*	0.224	*Ubiquitin*	5.63
7	*Ubiquitin*	0.94	*β-tubulin*	0.97	*Ubiquitin*	0.248	*eEF*	5.66
8	*eEF*	1.18	*GAPDH*	0.99	*GAPDH*	0.275	*GAPDH*	6.4
Best combination of two genes					*18S rRNA* and *eEF*	0.102		
**Plant organs (stem and leaf)**								
1	*18S rRNA*	1.39	*18S rRNA*	0.76	*18S rRNA*	0.125	*18S rRNA*	1
2	*25S rRNA*	1.48	*ACCase*	0.83	*ACCase*	0.146	*25S rRNA*	1.86
3	*Actin*	1.67	*25S rRNA*	0.89	*β-tubulin*	0.204	*Actin*	3.46
4	*Ubiquitin*	1.72	*Actin*	1.1	*Ubiquitin*	0.206	*ACCase*	3.76
5	*ACCase*	1.79	*GAPDH*	1.38	*25S rRNA*	0.214	*Ubiquitin*	4.36
6	*GAPDH*	2.02	*Ubiquitin*	1.51	*Actin*	0.214	*GAPDH*	5.73
7	*eEF*	2.37	*eEF*	1.81	*eEF*	0.229	*eEF*	7
8	*β-tubulin*	2.89	*β-tubulin*	3.01	*GAPDH*	0.323	*β-tubulin*	8
Best combination of two genes					*ACCase* and *eEF*	0.113		
**Drought stress (water-treated vs untreated)**								
1	*18S rRNA*	0.93	*ACCase*	0.26	*eEF*	0.191	*18S rRNA*	1.19
2	*β-tubulin*	1	*18S rRNA*	0.54	*β-tubulin*	0.295	*β-tubulin*	2
3	*25S rRNA*	1.05	*25S rRNA*	0.73	*18S rRNA*	0.304	*25S rRNA*	3
4	*ACCase*	1.21	*β-tubulin*	0.75	*ACCase*	0.326	*ACCase*	3.25
5	*Actin*	1.24	*Actin*	0.77	*GAPDH*	0.349	*Actin*	5
6	*GAPDH*	1.27	*GAPDH*	1.02	*25S rRNA*	0.406	*GAPDH*	5.42
7	*Ubiquitin*	1.35	*Ubiquitin*	1.17	*Actin*	0.516	*Ubiquitin*	6.74
8	*eEF*	2.3	*eEF*	1.48	*Ubiquitin*	0.581	*eEF*	8
Best combination of two genes					*GAPDH* and *18S rRNA*	0.165		
**All samples (plant life stages, plant organs and drought stress)**								
1	*18S rRNA*	1.39	*18S rRNA*	0.72	*18S rRNA*	0.175	*18S rRNA*	1
2	*25S rRNA*	1.48	*ACCase*	0.73	*ACCase*	0.198	*25S rRNA*	1.86
3	*Actin*	1.69	*25S rRNA*	0.8	*B-Tubulin*	0.237	*Actin*	3.22
4	*ACCase*	1.75	*Actin*	1.06	*eEF*	0.249	*ACCase*	3.36
5	*Ubiquitin*	1.97	*GAPDH*	1.33	*25S rRNA*	0.257	*Ubiquitin*	5.48
6	*GAPDH*	1.97	*Ubiquitin*	1.68	*Actin*	0.303	*GAPDH*	5.48
7	*eEF*	2.39	*eEF*	1.77	*Ubiquitin*	0.323	*eEF*	7
8	*β-tubulin*	2.78	*β-tubulin*	2.75	*GAPDH*	0.369	*β-tubulin*	8
Best combination of two genes					*Ubiquitin* and *ACCase*	0.142		

RefFinder compares the results evaluated by four different programs (comparative ΔCt method, geNorm, BestKeeper and NormFinder) and based on the geomean of ranking values, provides a comprehensive ranking. St. dev.: standard deviation; St. dev [+ /− CP]: standard deviation of crossing point (CP) values, eEF: eukaryotic elongation factor, ACCase: Acetyl-CoA carboxylase.

### Plant organs related studies

In gene expression studies with the plant organs, *18S rRNA* has been ranked as the most stable gene by comparative ΔCt, BestKeeper and RefFinder (Table 2). NormFinder analysis identified *ACCase* and eEF as the most stable genes (Table 2). Based on the geNorm analysis, *18S rRNA* and *ACCase* might be the best reference gene for plant organs-related studies in *B. sterilis* (Fig. 2). *β-TUB* was identified as the least stable gene by comparative ΔCt, BestKeeper and RefFinder, whereas NormFinder and geNorm analysis identified GAPDH as the least stable gene.

#### Under drought stress

For studies under drought stress, comparative ΔCt and RefFinder identified *18S rRNA* as the most suitable reference gene (Table 2). BestKeeper software identified *ACCase* as the most stable reference gene (Table 2). NormFinder analysis revealed *GAPDH* and *18S rRNA* as the most stable genes, whereas geNorm analysis ranked *18S rRNA* and *β-TUB* as the best reference gene (Table 2, Fig. 2). *eEF* was identified as the least stable gene by ΔCt, BestKeeper and RefFinder, whereas NormFinder and geNorm analysis identified *UBQ* as the least stable gene for studies under drought stress on *B. sterilis*.

#### Combined conditions

When all the conditions were taken together, *18S rRNA* was identified as the most stable reference gene, irrespective of the method (Table 2, Fig. 2). NormFinder analysis results for combined conditions revealed that *UBQ and ACCase* could be considered the best reference gene, and the geNorm algorithm ranked *18S rRNA* and *ACCase*, as the best reference gene (Table 2, Fig. 2). *β-TUB* was identified as the least stable gene by comparative ΔCt, BestKeeper and RefFinder, whereas NormFinder and geNorm analysis identified *GAPDH* as the least stable gene.

### Pairwise variation analysis

**Figure 3:**
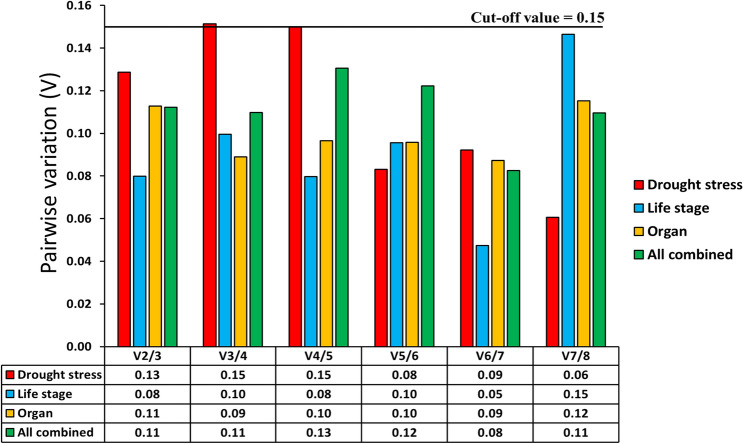
Pairwise variation to determine the optimal number of reference genes. The recommended cutoff value under which there is no need for another gene is 0.15.

The pairwise variation (Vn/Vn + 1) was calculated based on the geNorm algorithm. The optimal number of the reference genes required for the normalisation were determined from the pairwise variation results, based on the average expression stability (M) values (cutoff: M < 1.5). The optimal number of the reference genes required for the normalisation for experiments related to the developmental stages and plant organs are 1 and 2, respectively. However, to avoid any biases in the normalization, we recommend using 2 reference genes for developmental stages. Hence, we recommend using *18S rRNA* and *ACCase*, as housekeeping genes for developmental stages and plant organ-related studies in *B. sterilis*. Under drought stress, 4 candidate genes (*18S rRNA*, *β-TUB*, *25S rRNA* and *ACCase*) were considered suitable for normalisation. When all the conditions were considered together, the pairwise variation result suggested that 2 reference genes will be required for the normalisation (Fig. 3). Therefore, *18S rRNA* and *ACCase* were identified as the most suitable gene when all the conditions were considered together.

### Relative expression of the *acetolactate synthase* (*ALS*) gene under herbicide stress

**Figure 4:**
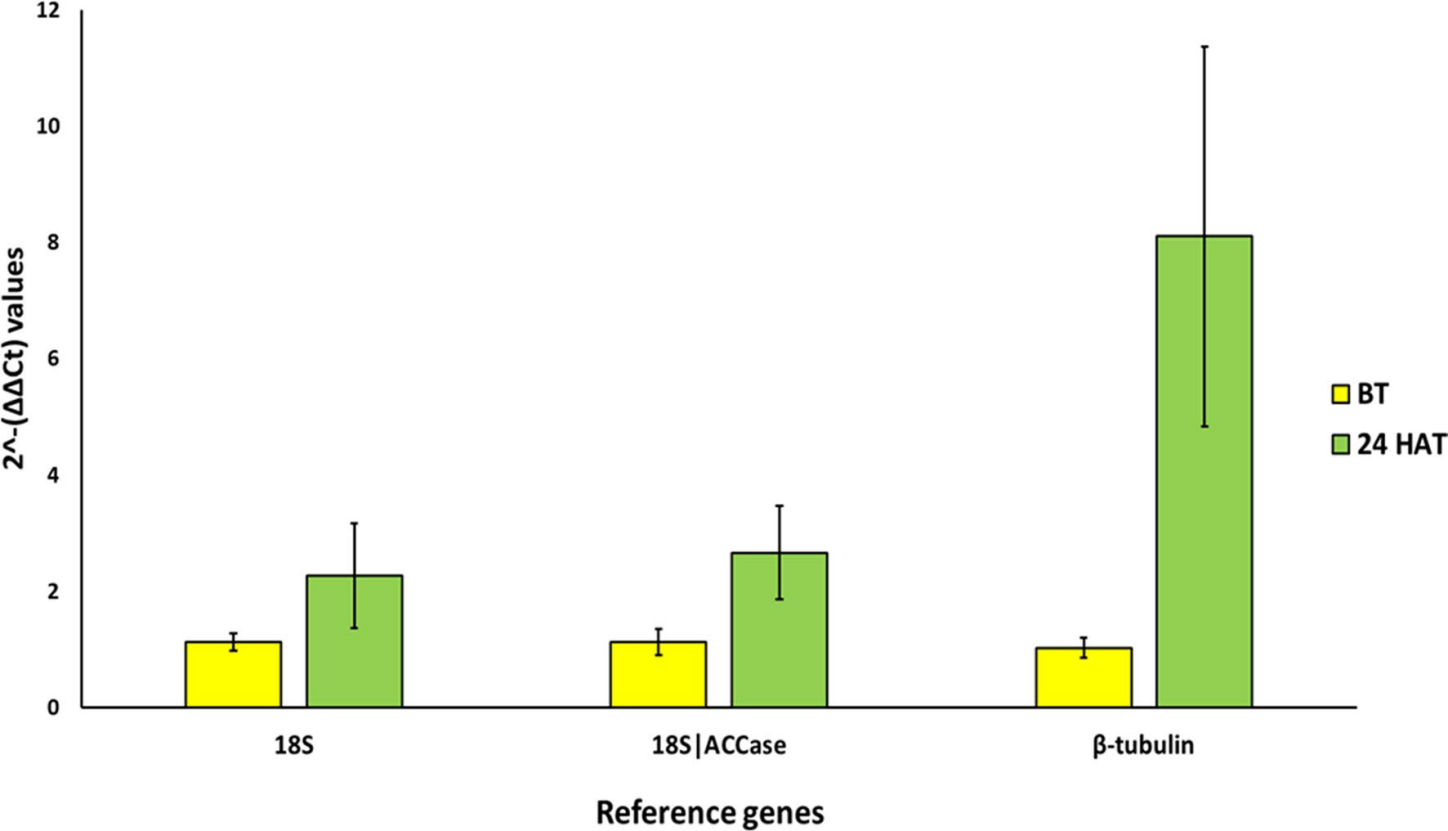
Relative expression of the acetolactate synthase gene under herbicide stress. Relative gene expression before herbicide treatment (BT) and 24 h after treatment (24 HAT) were compared, and normalization was done with *18S rRNA*, *18S rRNA*|*ACCase* and *β-tubulin*.

Based on the analysis of the commonly used software for reference gene analysis, *18S rRNA* and a combination of *18S rRNA* and *ACCase* were identified as the most suitable candidate genes for gene expression studies in *Bromus sterilis,* whereas *β-TUB* as the most unstable gene. To validate the reliability of the candidate genes, relative expression of the *acetolactate synthase* under herbicide stress was evaluated using the best and the least stable candidate genes. When normalised with *18S rRNA* and a combination of 18S rRNA and ACCase, *B. sterilis* biotype showed twofold *ALS* gene overexpression after herbicide treatment compared to the control, whereas with *β-TUB*, the result is almost eight times (Fig. 4).

## Discussion

Recent reports from the United Kingdom and the Czech Republic on *B. sterilis*, developing resistance against commonly used herbicides, indicate that if they remained uncontrolled, these species might become a concern worldwide^25,26^. Herbicide resistance mechanisms can be target-site based (TSR) and/or non-target site-based (NTSR). Target-site based mechanisms involve nucleotide polymorphisms^27^, gene amplification^6^ and gene over-expression^28^, whereas increased detoxification by enhanced metabolism^29,30^ and/or reduced herbicide uptake and translocation^29^ fall under non-target site-based herbicide resistance. Irrespective of the mechanism/s of resistance, qRT-PCR and the next-generation sequencing technologies have been used recently as a common technique to investigate the resistance mechanism in different weed species^5^. qRT-PCR experiments require an appropriate reference gene to normalise the target transcript levels. Any misapprehension in selecting a stable reference gene might lead to ambiguous results. Hence, the selection of a reliable reference gene is obligatory. Even though suitable candidate genes under different experimental condition were identified in many weedy species, like *Alopecurus sp.*^20^, *Eleusine* sp.^8^, *Avena* sp.^31,32,33^, *Descurainia* sp.^34^ etc., but to date, there are no reports on the systematic selection of stable reference genes under any conditions for barren brome or any other related brome species.

This study used qRT-PCR to evaluate the expression stability of eight candidate reference genes in barren brome under different experimental conditions. The most stable reference genes for each experimental condition were identified exclusively. geNorm software identified the ideal pair of genes with the minor variation in their expression ratios for each experimental condition. For studies related to life stages, geNorm identified that combining two reference genes would be suitable for normalising the qRT-PCR based gene expression values. *18S rRNA and ACCase* was chosen as the best reference gene for the studies with life stages of *B. sterilis*. For studies related to plant organs and under drought stress, pairwise variation analysis recommended using two and four genes, respectively. *18S rRNA* and *ACCase* were chosen as the most suitable candidates for plant organs-related studies, whereas, for studies under drought stress, we recommend using *18S rRNA*, *β-TUB*, *25S rRNA* and *ACCase*. When all the conditions were considered together, *18S rRNA* and *ACCase* were identified as the most suitable gene. Validation under herbicide stress indicated that both *18S rRNA* and the combination of *18S rRNA* and *ACCase* could be suitable. *18S rRNA*, a component of the 40S ribosomal small subunit in eukaryotes, has been recognised to have a steady expression in grasses under different stresses in earlier studies^35,36^. 18S rRNA is a primary constituent of all eukaryotic cells. Hence, *18S rRNA* is known to have extremely high expression in most cell types, so it can be challenging to use it as an endogenous normaliser gene. Moreover, synchronized use of multiple reference genes will also decrease the chance of biased normalisations. Finally, from our study results, *18S rRNA* and *ACCase* appeared to be the most suitable reference genes to normalise the qRT-PCR data in *B. sterilis*.

Rapid advances in molecular biology techniques in plant biotechnology have increased the demand for identification of reference genes, which will be more stable than the traditional reference genes. The reference genes identified and validated in our study will assist the studies related to the elucidation of abiotic stress and its regulatory mechanisms. Comparative RNA-seq transcriptome analysis between the control and experimental plants can be regarded as the most straightforward way to identify the genes involved in abiotic stresses like herbicide stress^20^. Recent studies on herbicide resistance mechanisms of *B. sterilis* suggest that both TSR and NTSR can be linked with the herbicide resistance in these species^25,26^. Nevertheless, detailed follow-up studies are essential to delineate further the regulatory mechanisms underlying the observed herbicide resistance mechanism^24^. However, among the herbicide resistance mechanisms, NTSR mechanisms are considered more complex to elucidate than the TSRs^20^. Comparative RNA-seq studies between the herbicide-resistant and susceptible plants will facilitate unravelling plausible resistance mechanisms in barren brome. Nevertheless, the RNA-seq data should be further cross-checked via qPCR, whose reliability depends on selecting the reference genes. This is the first study to evaluate and validate experiment-condition specific reference genes in brome species to the best of our knowledge. We had identified and validated internal reference gene suitable for normalising qRT-PCR experiments. Thus, our reference genes can be used during any RNA-seq based transcriptome or gene expression studies on *B. sterilis*. Our findings provide a basis for future molecular work on *B. sterilis* and can also be used during gene expression studies in other related species after preliminary validation.

## Methods

### Plant materials

A single population of *B. sterilis*, used for this study. *B. sterilis* was collected from winter wheat fields in the Ústecký region of the Czech Republic (50.2612525 N, 13.4818572 E). *Bromus sterilis* is an undesirable arable weed, so there are no specific country regulations for manipulation with it. No permissions were necessary to collect plant samples. 25 cm^2^ pots (filled with chernozem soil, clay content 46% (loamy soil), soil pH (KCl) 7.5, sorption capacity of soil: 209 mmol (+), 87 mg kg^−1^ P, 203 mg kg^−1^ K, 197 mg kg^−1^ Mg, 8073 mg kg^−1^ Ca), were used to plant the seeds. The pots were kept in an open‐air vegetation hall (with roof-top). Plant samples from three developmental stages (2-leaves stage, 3-leaves stage and 4-leaves stage), two different plant organs (shoots and leaves) and one abiotic stress (drought stress) were used for this study. For drought stress, watering was interrupted when the plants reach the three to four leaves stage, till symptoms of wilting were observed. Wilted leaves samples were collected and stored at − 80 °C (until further use). For herbicide stress, the plants were treated with pyroxsulam (a group of triazolopyrimidine sulfonamide ALS-inhibiting herbicide) at two to three leaf stage with recommended dose (1.875 g a.i. ha^–1^). Herbicide was sprayed using a laboratory spray chamber equipped with a Lurmark 015F80 nozzle with a spray volume of 250 L ha^–1^ and pressure 120 kPa. The leaves samples were collected before treatment and 24 h after treatment and stored at − 80℃ for RNA extraction. All experiments conducted in this study, including the collection of plant material, are in compliance with relevant institutional, national, and international guidelines and legislation.

### RNA extraction, complementary DNA (cDNA) synthesis and primer design

RNeasy Mini Kit (Qiagen, Hilden, Germany) was used to extract RNA from the fresh tissues (± 80 mg per sample). TURBO DNA-free (Invitrogen, US) Kit was used to remove gDNA contamination. RNA integrity was verified by running the samples on 1.2% agarose gel electrophoresis. cDNA was synthesised by High Capacity cDNA Reverse Transcription Kit (Applied Biosystems, USA) from the quality-checked gDNA-free RNA template (1 μg per reaction). Degenerate primers were designed for eight common candidate reference genes (*UBQ*, *ACT*, *GAPDH*, *18S rRNA*, *25S rRNA*, *ACCase*, *β-TUB* and *eEF*) based on their homologous sequences in other plants species (Table 1). The primers were designed using Primer-BLAST (https://www.ncbi.nlm.nih.gov/tools/primer-blast/) and Primer3 software (https://bioinfo.ut.ee/primer3-0.4.0/). All the primers were tested by general PCR, performed using a C1000 thermocycler (Bio‐Rad, Hercules, CA, USA), using cDNA template (10 ng per reaction). The thermocycler was programmed at an initial denaturation step at 95 °C for 5 min, followed by 40 cycles of 5 s at 95 °C, 10 s at 57 to 60 °C (based on the annealing temperature of the primer pairs), and 30 s at 72 °C along with a final extension step for 10 min at 72 °C. The PCR amplified products were verified in the 1.5% agarose gel electrophoresis (data not shown).

### qRT-PCR experiment and data analysis

PowerUp SYBR Green Master Mix (Applied Biosystems, USA) was used to conduct qRT-PCR assay in StepOne™ Real-Time PCR System (Applied Biosystems, USA). The reaction mixture contained 5 μL of SYBR Green Master Mix, 1 μL of primer mix and 4 μL of cDNA (2.5 ng μL^−1^). For primer efficiency (E) and correlation coefficient (R^2^) calculation, qRT-PCR assay was performed with diluted series of cDNA samples. E = {10^(−1/slope) ^− 1 * 100%} was used to calculate the values of E. The thermocycler was programmed at an initial denaturation step at 95 °C for 10 min, followed by 40 cycles of 15 s at 95 °C and 1 min at 57 to 60 °C (based on the annealing temperature of the primer pairs). To obtain the melting curves, stepwise heating was performed from 60 to 95 °C. All qRT-PCR experiments were conducted with 5 biological replicates. Quantification cycle threshold (Ct) values obtained from the StepOne Real-Time PCR System (Applied Biosystems, USA) was exported and used for further calculations. Gene expression stabilities of the eight candidate genes in the *B. sterilis* were examined by geNorm, NormFinder and BestKeeper, according to Chen et al.^8^. Besides, comparative ΔCt^37^ and RefFinder^8^ were also used. Before and after herbicide treatment, the relative ALS gene expression was calculated using the 2^−ΔΔCt^ method^38,39^. NormFinder software estimates the intra- and intergroup variation. These variations are then combined into a stability value. The gene with minimal variation is ranked as the best by the software. geNorm program estimates an expression stability value (M) for each gene. Genes with the lowest M values have the most stable expression. BestKeeper ranks the candidate genes based on standard deviation values of cycle threshold (Ct) or crossing point values (CP) and coefficient of correlation (r) values. A gene with a standard deviation value below 1 and a coefficient of correlation value close to 1 is considered to have more stable gene expression than others. RefFinder integrates the available well-known programs for reference gene screening (geNorm, NormFinder, BestKeeper, and the comparative Delta-Ct method) and calculates the geometric mean of ranking values to give the overall ranking. The genes with a minimal geometric mean of ranking values are categorized as the best^37^.

### Ethical approval

No permissions were necessary to collect plant samples. All experiments conducted in this study, including the collection of plant material, are in compliance with relevant institutional, national, and international guidelines and legislation.

### Ethics statement

This article does not contain any studies with human or animal subjects.

## Supplementary Information

Supplementary Figures.
