# Supplementary material for: Identification of the most suitable reference gene for gene expression studies with development and abiotic stress response in Bromus sterilis
Source: Sci Rep. 2021 Jun 28;11:13393. doi: 10.1038/s41598-021-92780-1 (PMC8238991; doi:10.1038/s41598-021-92780-1)
Supplement: Supplementary file 1 — Supplementary Figures. [file 41598_2021_92780_MOESM1_ESM.docx]

**Identification of the most suitable reference gene for gene expression studies with development and abiotic stress response in *Bromus sterilis***

Madhab Kumar Sen^a^, Kateřina Hamouzová^a^, Pavlina Košnarová^a^, Amit Roy^b#^, Josef Soukup^a*#^

**Short title:** Reference genes for molecular studies in barren brome

^a^Department of Agroecology and Crop Production, Faculty of Agrobiology, Food and Natural Resources, Czech University of Life Sciences Prague, Kamýcká 1176, Prague 6, 165 00, Suchdol, Czech Republic.

^b^Faculty of Forestry and Wood Sciences, EXTEMIT-K and EVA.4.0 Unit, Czech University of Life Sciences, Kamýcká 1176, Prague 6, 165 00, Suchdol, Czech Republic.

***Address correspondence to**:

Josef Soukup

Department of Agroecology and Crop Production, Faculty of Agrobiology, Food and Natural Resources, Czech University of Life Sciences Prague, Kamýcká 1176, Prague 6, 165 00, Suchdol, Czech Republic. Email: [soukup@af.czu.cz](mailto:soukup@af.czu.cz).

# indicates that the authors had contributed equally


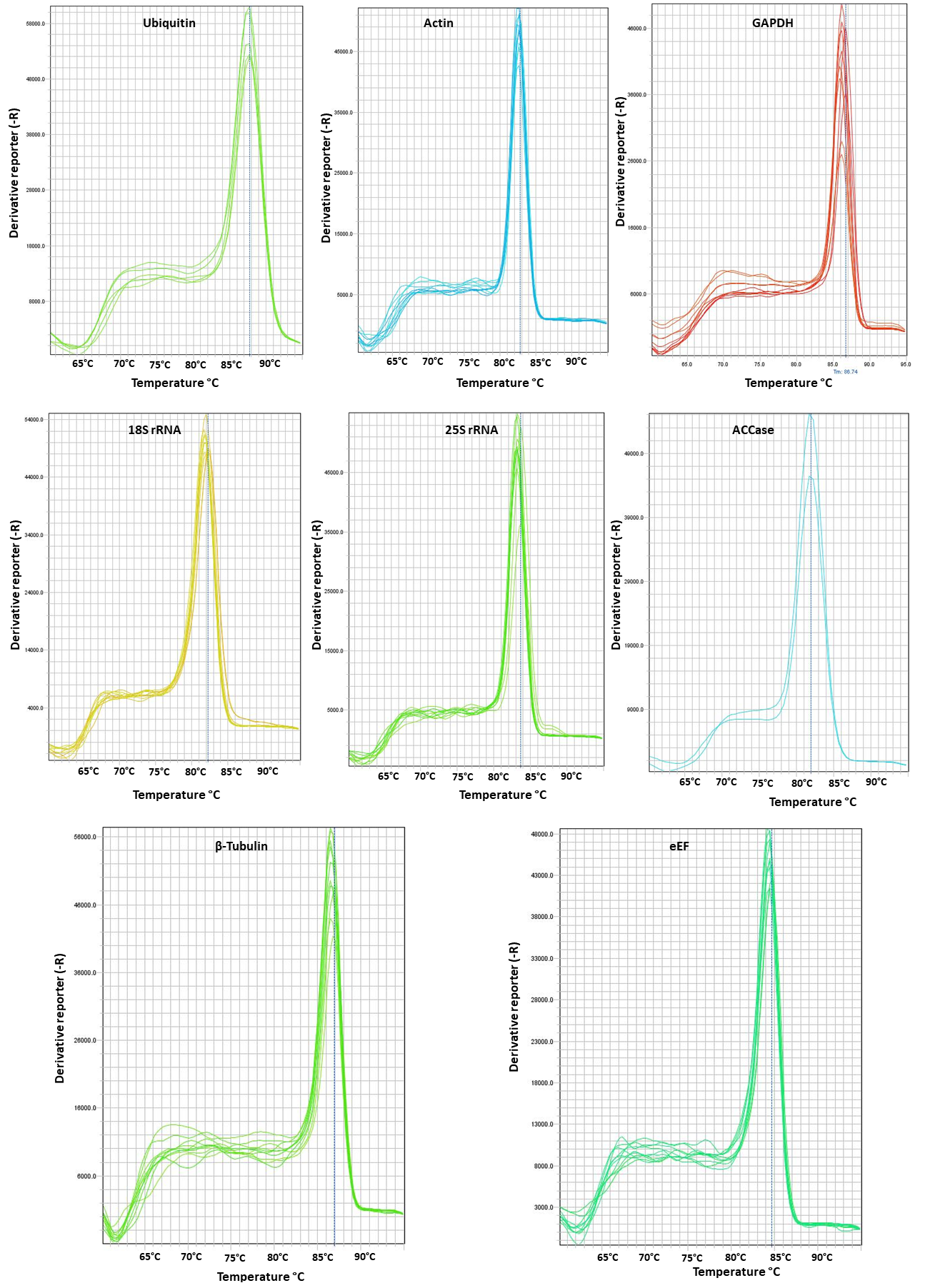


**Supplementary Figure S1**: Melt curve analyses of the eight candidate reference genes.


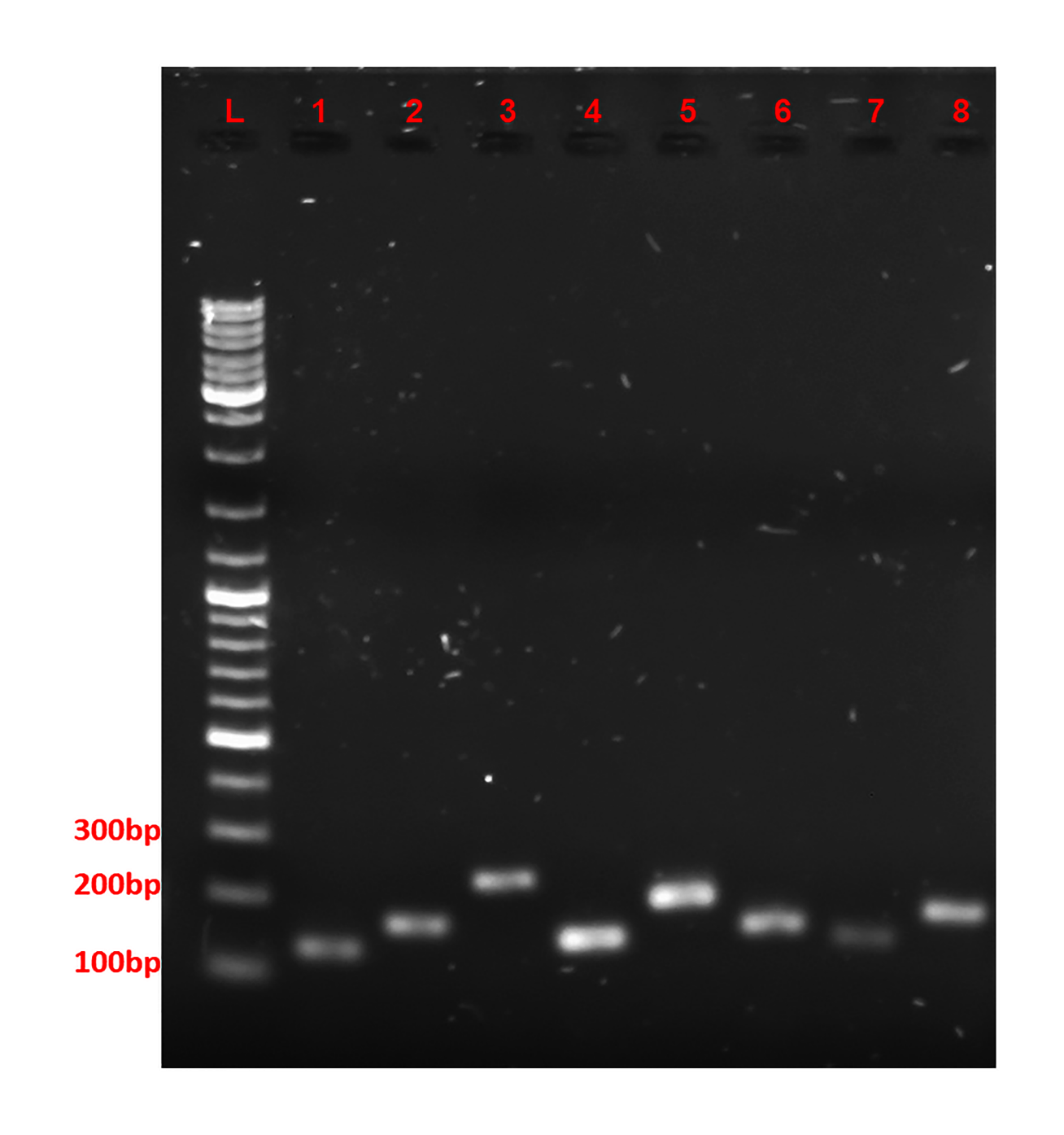


**Supplementary figure 2:** Gel picture (1.5% agarose gel) showing the PCR amplification specificity of the candidate reference genes. Amplicon band is shown for *Ubiquitin, Actin, GAPDH, 18S rRNA, 25S rRNA, ACCase, β-Tubulin* and *eEF* (1 to 8, respectively, in the picture). Gene ruler DNA Ladder Mix (Fermentas, US) was used to identify the product size.
